# Supplementary figures and images for: Differential PARP inhibitor responses in BRCA1-deficient and resistant cells in competitive co-culture
Source: PLoS One. 2025 Sep 22;20(9):e0332860. doi: 10.1371/journal.pone.0332860 (PMC12453244; doi:10.1371/journal.pone.0332860)

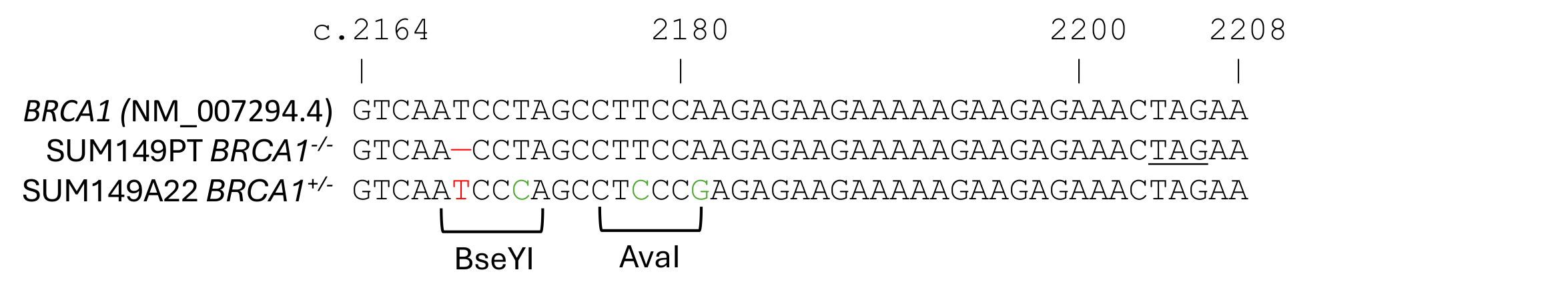

Supplement: S1 Fig — In red is where the c.2169delT mutation is and subsequent correction mutation. In green are silent mutations which create new BseYI and AvaI restriction sites that can be used for genotyping the SUM149.A22 correction. (TIFF) [file pone.0332860.s001.tiff]

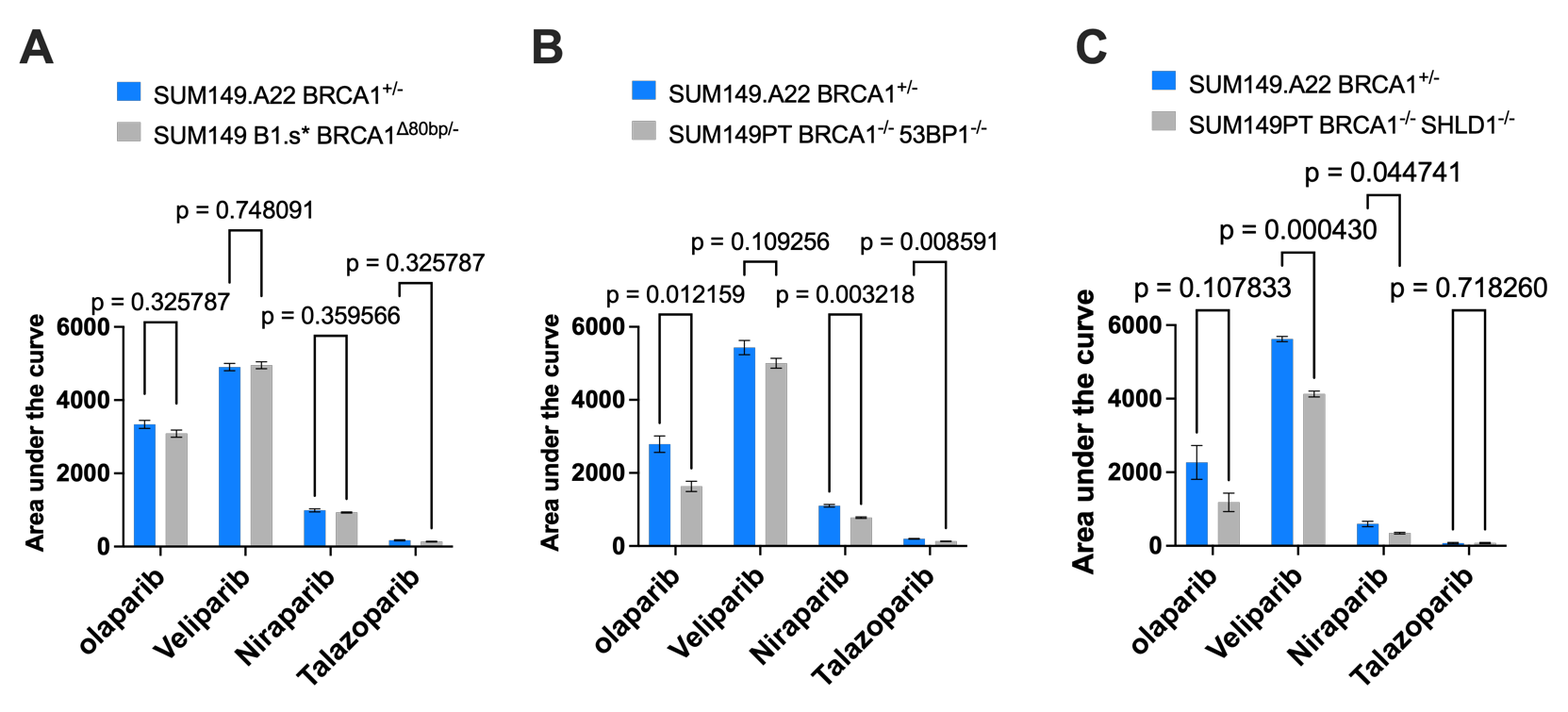

Supplement: S2 Fig — (A) – SUM149.A22 vs SUM149 B1.s*, (B) SUM149.A22 vs SUM149 53 BP1 and (C) SUM149.A22 vs SUM149 SHLD1). These plots are derived from the data in Fig 4, in which they show statistical significance between the survival curves of the cell lines in the competitive growth assay. Unpaired t-tests were performed between each isogenic cell pair in each treatment. (TIFF) [file pone.0332860.s002.tiff]

RPE BRCA1<sup>+/-</sup>  
RPE BRCA1<sup>-/-</sup>  
SUM149PT  
SUM149.A22  
SUM149 B1.S\*

250 kDa -

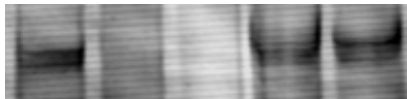

anti-BRCA1

250 kDa -

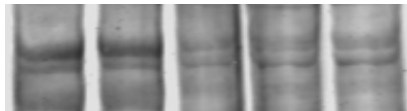

Total protein stain

Supplement: S3 Fig — Western blot was performed on 40 µg of cell lysate from RPE BRCA1+/+, RPE BRCA1-/-, SUM149PT, SUM149.A22 and SUM149 B1.s*. The membranes were stained with Revert™ 700 total protein stain as a loading control. (PDF) [file pone.0332860.s003.pdf]
