# Supplementary material for: Differential PARP inhibitor responses in BRCA1-deficient and resistant cells in competitive co-culture
Source: PLoS One. 2025 Sep 22;20(9):e0332860. doi: 10.1371/journal.pone.0332860 (PMC12453244; doi:10.1371/journal.pone.0332860)
Supplement: S1 Table — (DOCX) [file pone.0332860.s004.docx]

| Cell Line | Tissue | *BRCA1* variants | Notable gene variants | Reference |
| --- | --- | --- | --- | --- |
| SUM149PT | Breast cancer ductal carcinoma | *BRCA1* c.2169delT |  | (16,28) |
| SUM149.A22 | Breast cancer ductal carcinoma | Reversion of *BRCA1* c.2169delT |  | This study |
| SUM149 B1.s* | Breast cancer ductal carcinoma | *BRCA1* c.2169delT; c.2175-2254delC-T |  | (16) |
| SUM149 *SHLD1* | Breast cancer ductal carcinoma | *BRCA1* c.2169delT | *SHLD1*  KO | (14) |
| SUM149 *53BP1* | Breast cancer ductal carcinoma | *BRCA1* c.2169delT | *53BP1 KO* | (14) |
| RPE *BRCA1*^+/+^ | Retinal pigment epithelium |  | *P53* KO | (29) |
| RPE BRCA1^-/-^ | Retinal pigment epithelium | c.1346delA; c.1345delC | *P53* KO | (29) |

S1 Table. Background information on cell lines used in the competitive growth assays.
