# Supplementary material for: Differential PARP inhibitor responses in BRCA1-deficient and resistant cells in competitive co-culture
Source: PLoS One. 2025 Sep 22;20(9):e0332860. doi: 10.1371/journal.pone.0332860 (PMC12453244; doi:10.1371/journal.pone.0332860)
Supplement: S2 Table — (DOCX) [file pone.0332860.s005.docx]

S2 Table. Summary of IC50’s and area under the curve figures for each of the competitive growth assays.

| **Cell combination** | **Compound** | **IC50 (µM)** | **AUC** |
| --- | --- | --- | --- |
| SUM149PT vs SUM149.A22 | Olaparib | 1.08 vs 5.23 | 166.2 vs 1622.0 |
|  | Veliparib | 1.34 vs >50 | 1404 vs 3455 |
|  | Niraparib | 0.67 vs 3.91 | 166.6 vs 671 |
|  | Talazoparib | 0.05 vs 0.48 | 16.91 vs 104.2 |
| RPE *BRCA1*^-/-^ vs RPE *BRCA1*^+/+^ | Olaparib | 0.1 vs 12.96 | 79.36 vs 1404 |
|  | Veliparib | 3.16 vs >50 | 1081 vs 6001 |
|  | Niraparib | 0.45 vs 12.31 | 565.3 vs 1711 |
|  | Talazoparib | 0.01 vs 0.97 | 62.94 vs 257.5 |
| SUM149PT vs SUM149 B1.s* | Olaparib | 1.99 vs 47.60 | 375.6 vs 4333 |
|  | Veliparib | 41.90 vs > 50 | 3545 vs 6311 |
|  | Niraparib | 1.45 vs 8.55 | 212.9 vs 977.5 |
|  | Talazoparib | 0.08 vs 0.43 | 29.5 vs 129.7 |
| SUM149.A22 vs SUM149 B1.s* | Olaparib | 36.46 vs 34.08 | 3339 vs 3085 |
|  | Veliparib | >50 vs >50 | 4904 vs 4953 |
|  | Niraparib | 9.54 vs 9.34 | 990.4 vs 932 |
|  | Talazoparib | 0.85 vs 0.453 | 170.8 vs 136.4 |
| SUM149PT vs SUM149 *53BP1* | Olaparib | 0.96 vs 24.54 | 179.3 vs 3460 |
|  | Veliparib | 24.37 vs >50 | 2549 vs 6855 |
|  | Niraparib | 0.91 vs 4.64 | 129.2 vs 793.4 |
|  | Talazoparib | 0.08 vs 0.75 | 17.4 vs 204.2 |
| SUM149.A22 vs SUM149 *53BP1* | Olaparib | 18.52 vs 11.32 | 2789 vs 1633 |
|  | Veliparib | >50 vs >50 | 5433 vs 5002 |
|  | Niraparib | 10.67 vs 7.65 | 1103 vs 777.1 |
|  | Talazoparib | 1.13 vs 0.75 | 199.2 vs 130.8 |
| SUM149PT vs SUM149 *SHLD1* | Olaparib | 1.89 vs 13.65 | 249.3 vs 2508 |
|  | Veliparib | 33.55 vs >50 | 3325 vs 6087 |
|  | Niraparib | 1.01 vs 4.39 | 131.5 vs 738.2 |
|  | Talazoparib | <0.079 vs <0.152 | 21.7 vs 266.1 |
| SUM149.A22 vs SUM149 *SHLD1* | Olaparib | 14.06 vs 4.76 | 2267 vs 1184 |
|  | Veliparib | >50 vs >50 | 5621 vs 4127 |
|  | Niraparib | 4.26 vs 2.74 | 596.3 vs 345.8 |
|  | Talazoparib | 0.25 vs 0.132 | 73.8 vs 75.4 |
