# Supplementary material for: Differential PARP inhibitor responses in BRCA1-deficient and resistant cells in competitive co-culture
Source: PLoS One. 2025 Sep 22;20(9):e0332860. doi: 10.1371/journal.pone.0332860 (PMC12453244; doi:10.1371/journal.pone.0332860)
Supplement: S3 Table — (DOCX) [file pone.0332860.s006.docx]

S3 Table. SUM149 cell line doubling times.

| **Cell line** | **SUM149PT *BRCA1^-/-^*** | **SUM149.A22 *BRCA1^+/-^*** | **SUM149 B1.s* *BRCA1^Δ80bp/-^*** | **SUM149 *BRCA1^-/-^53BP1^-/-^*** | **SUM149 *BRCA1^-/-^SHLD1^-/-^*** |
| --- | --- | --- | --- | --- | --- |
| Doubling time | 26.5 h | 27.4 h | 27.1 h | 30.1 h | 31.7 h |
