# Supplementary material for: Differential PARP inhibitor responses in BRCA1-deficient and resistant cells in competitive co-culture
Source: PLoS One. 2025 Sep 22;20(9):e0332860. doi: 10.1371/journal.pone.0332860 (PMC12453244; doi:10.1371/journal.pone.0332860)
Supplement: S2 Data — (PPTX) [file pone.0332860.s008.pptx]

## Slide 1
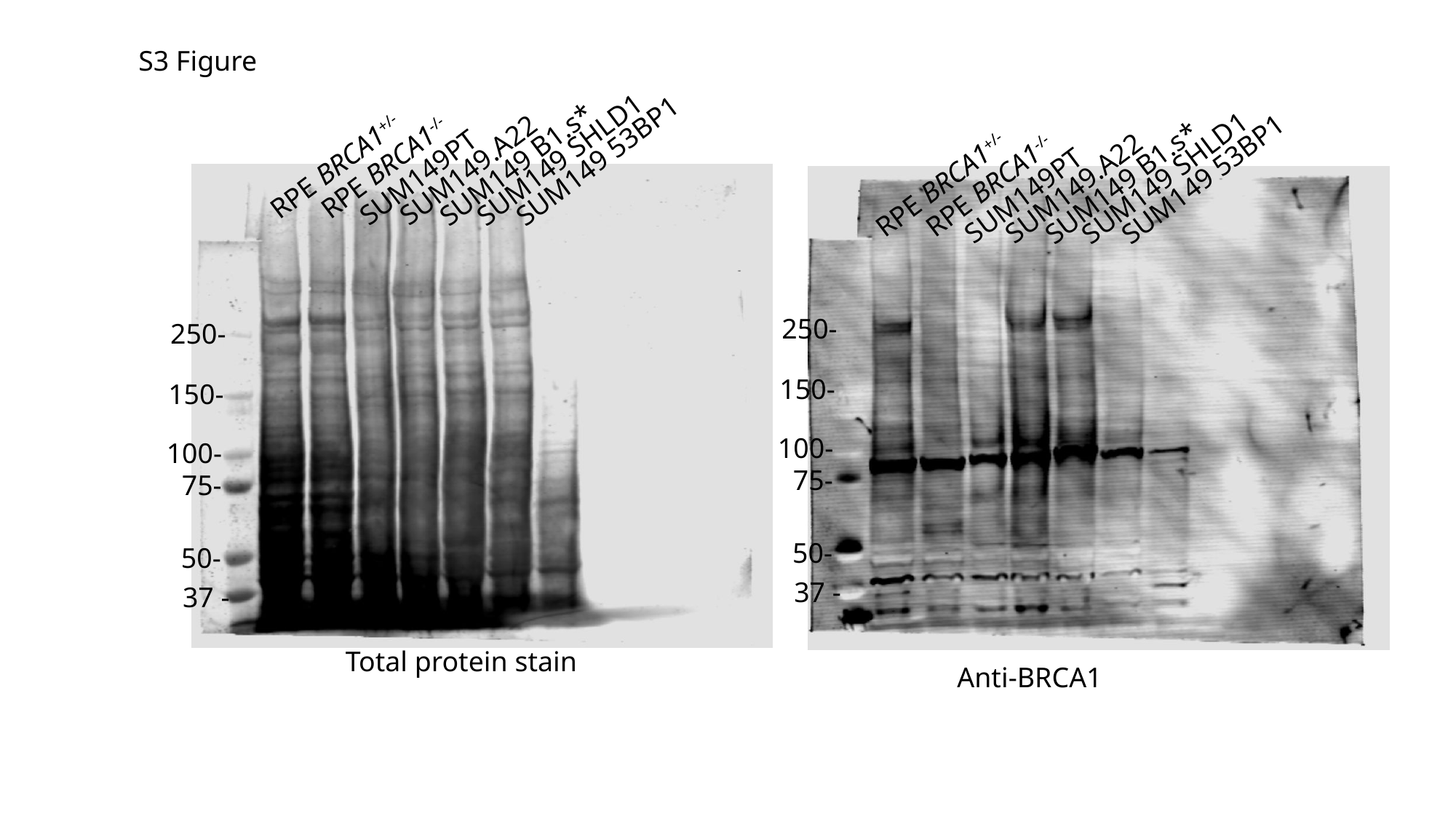

S3 Figure
SUM149 SHLD1
SUM149 53BP1
SUM149 B1.s*
RPE BRCA1+/-
RPE BRCA1-/-
SUM149.A22
SUM149PT
SUM149 SHLD1
SUM149 53BP1
SUM149 B1.s*
RPE BRCA1+/-
RPE BRCA1-/-
SUM149.A22
SUM149PT
250-
250-
150-
150-
100-
100-
75-
75-
50-
50-
37 -
37 -
Total protein stain
Anti-BRCA1
