# Supplementary material for: Differential PARP inhibitor responses in BRCA1-deficient and resistant cells in competitive co-culture
Source: PLoS One. 2025 Sep 22;20(9):e0332860. doi: 10.1371/journal.pone.0332860 (PMC12453244; doi:10.1371/journal.pone.0332860)
Supplement: Attachment — Submitted filename: Soetomo et al PLOS ONE review report.docx [file pone.0332860.s009.docx]

Inhibition of PARP1/2 (PARPi) in homologous recombinant (HR) repair deficient cells is a classic example of synthetic lethality. Here, the authors focused on describing their competitive co-culture assay in the context of BRCA1-deficiency and PARPi. Specifically, they compared the fluorescent readouts in isogenic cell lines of BRCA1-deficient (mCherry) and BRCA1-proficient (eGFP) cells that are co-cultured in the presence of different concentrations of PARPi. Soetomo et al also applied their competitive assay in other previously reported PARPi resistant BRCA1 mutant/deficient clones (Azurite reporter). Indeed, BRCA1-deficient cells are sensitive to the PARPi tested (olaparib, niraparib, veliparib and talozoparib) while PARPi-resistant cell lines showed subtle differences compared to BRCA1-proficent cells. The study design is well executed with the data figure well assembled and easy to be understood. The statistical analysis is also clear.

**The manuscript by virtue of its results is a solid candidate for publication if the authors can address the following concerns:**

1. **Lack of western blot to confirm the presence or absence of mutant proteins.**

The authors generated SUM149.A22. that is a BRCA1+/- heterozygous cell line. Additionally, the study also utilized PARPi-resistant clones SUM149 B1.s* BRCA1^D80bp/-^ that restored the reading frame of the mutant allele in SUM149 cells. Both cell lines behave similarly in terms of PARPi resistance. Deletion of either 53BP1 or SHLD1 also restored PARPi resistance.

A western blot analysis for all the cell lines used (SUM149 and RPE) is helpful to determine the BRCA1 protein levels in the BRCA1-revertant and mutant cell lines used. After all, PARPi resistance is highly correlated with BRCA1 protein levels.

2. **How is the cell growth of the cells in the absence of PARPi considering that rapidly proliferating cells may be easier to be targeted with PARPi?**

I wonder if the revertant (SUM149.A22., SUM149 B1.s* )or PARPi-resistant (SUM149 BRCA1^-/-^ 53Bp1^-/-^ and SUM149 BRCA1^-/-^ SHLD1^-/-^ )cell lines have any significant growth differences with the parental cell that is BRCA1-deficient.

Since this is a competitive growth assay over the course of several days, it will be important to determine if the cell lines have similar proliferation rates or proliferation rate of the cells are not a determining factor in their assay. The authors also acknowledge how interpretation of competitive growth assay can be context specific, citing how cells with higher proliferative rates are more sensitive to cisplatin (Reference 23)

3. **A table will be useful to summarize the IC50 values and AUCs with the appropriate statistical analysis of the different cell lines when treated with different PARPi.**
4. **How would the sensitivity profile be if the 53BP1 KO cells are co-cultured with SHLD1 KO cells? Can this experiment be done?**

The 53BP1-RIF1-SHLD1 complex is important for restricting DSB resection and promoting NHEJ, especially in BRCA1-deficient cells.

Results from Figure 4D, 4F, S2B, S2C is quite interesting as marginal sensitivity was observed in SUM149 BRCA1-/- 53Bp1-/- and SUM149 BRCA1-/- SHLD1-/- compared to SUM149.A22. This piece of result was mentioned in line 289-296 but not well expanded. What could be the reason behind the disparity between 53BP1-KO and SHLD1-KO when treated with different PARPis.

5. Minor concerns:
a) Typo line 193-194 for figure 2 legend “ The IC50’s in the SRB assay were 0.11 μM and 9.01 μM for the BRCA1+/- and BRCA1+/- cells “ . It should be BRCA1-/- with 0.11 μM IC50?
b) Word choice line 208-209 “ a phenomenon was observed” ; the word phenomenon is quite out of place in the long sentence.
