# Supplementary material for: Differential PARP inhibitor responses in BRCA1-deficient and resistant cells in competitive co-culture
Source: PLoS One. 2025 Sep 22;20(9):e0332860. doi: 10.1371/journal.pone.0332860 (PMC12453244; doi:10.1371/journal.pone.0332860)
Supplement: Attachment — Submitted filename: Review_PONE-D-25-26053.docx [file pone.0332860.s010.docx]

**Recommended suggestions and corrections**

Great work with insightful perspective on the effects of PARPi on BRCA1 resistant and deficient cells in co culture! The methodology is particularly well structured. Just a few minor suggestions from my end.

1) In the following section, I believe there should not be a comma if you are referring to a second complementary gene/pathway “By targeting a second, complementary gene or pathway that is synthetic lethal with the cancer-specific mutation, specific inhibitors can selectively kill cancer cells while sparing healthy cells lacking the mutation”
2) Line 39 – “PARPis” should be replaced with “PARPi” as the initially mentioned complete form of PARPi is “poly ADP-ribose polymerase inhibitors” (plural form)
3) Line 51 – “complexity that can be more closely represent in vivo environment” – the highlighted word should be removed
4) Line 61 – “In this present study,” replace highlighted word to “the” and change “competitive growth system that could be used” the highlighted word to “can” (if validated) or “may” (if requires further validation)
5) Line 62 – “PARPis” to “PARPi” (replace throughout the paper)
6) Line 100 – “After 48 h and 72 h after transfection,” change the highlighted word to “following”
7) Line 116 – “72 h after the first dose of drug, cells were drugged again with the same concentrations” change to “Following a 72 h incubation period, cells were again exposed to the same concentration of the drug” (also add what condition the cells were incubated in – e.g room temp or incubator)
8) Line 117 – “were fixed with with” remove duplicates
9) Line 125 – “The data was normalized using by calculating - normalized percentage of survival” remove highlight word and add “the” to the highlighted dash inserted
10) Line 128 – “A semilog curve was fitted to the data sets in which IC50’s were calculated from” replace with “A semi log curve was fitted to the data sets and IC50 values were calculated”
11) Line 132 – “PARPis one day and 4 days” - keep format of days consistent – 1 day and 4 days/one day and four days
12) Line 136 “The data was normalised using by calculating - normalized percentage of survival” remove highlighted word, add “the” to the highlighted dash inserted
13) Line 142-144 – “The SUM149PT cell line was originally derived from a breast adenocarcinoma and 143 contains a homozygous BRCA1 pathogenic variant, c.2169delT, which results in a frameshift 144 that leads to a premature stop codon (p.P724Lfs*12).” – part of the section uses past tense while part of it uses present tense. I would suggest using past tense as the rest of the paragraph is also in past tense and change “contains” to “contained”, “results” to “resulted” and “leads” to “led”
14) Line 156 – “The schematics of each assay is” change to “are”
15) Line 163 – “Fig 1. Schematic of the competitive growth assay vs SRB assay” and a full stop at the end
16) Line 164 in Fig 1 legend – “in the small wells of a microplate” change this to the actual 96-well plate, this sound informal
17) Fig 1 legend – present tense used again while the rest of paper is in past tense.
18) Fig 2 legend – “8 technical replicates were performed, and 192 error bars represent standard error of the mean” – I would recommend adding this section as the last sentences of the figure legend where you could write “Data is plotted as mean ± SEM where n = 8 for each treatment group”

Note: I am wondering why some of the data in this figure for both strains do not have error bars?

19) Fig 3 – “. A) SUM149PT BRCA1-/- vs SUM149.A22 BRCA1+/- 216 cells” could mention that is shows cell survivial. Same comment for “C) RPE BRCA1-/- vs RPE BRCA1+/+ 218 .”

Note: figures in (A) are showing error bars while figures in (C) are not. Could also mention the n number and whether the data represents mean +- SEM.

20) Figure 4 – same comment about the error bars – would recommend standardizing the error bars across figures.
21) Line 282 – “50% survival observed - BRCA1 negative cells” - add “for” in the highlighted dash position
22) Line 283-284 – “near-complete cells death” - make it singular
