# Supplementary material for: Differential PARP inhibitor responses in BRCA1-deficient and resistant cells in competitive co-culture
Source: PLoS One. 2025 Sep 22;20(9):e0332860. doi: 10.1371/journal.pone.0332860 (PMC12453244; doi:10.1371/journal.pone.0332860)
Supplement: Attachment — Submitted filename: Response to reviewers.docx [file pone.0332860.s012.docx]

**19^th^ August 2025**

**Dear Dr Zu Ye,**

Thank you for the opportunity of submitting a revised version of our manuscript entitled "Differential PARP inhibitor responses in BRCA1-deficient and resistant cells in competitive co-culture,". Overall, the reviewers were quite positive about the manuscript both in terms of rigour and presentation. E.g. Reviewer 1: “The system is well validated using both cancerous and non-cancerous isogenic cell lines, and the inclusion of resistance models….adds significant value to the findings.” “The methods are clearly described and technically sound.” “The conclusions are well supported by the data.”; Reviewer 3: “The study design is well executed with the data figure well assembled and easy to be understood. The statistical analysis is also clear. The manuscript by virtue of its results is a solid candidate for publication…”; and Reviewer 4 “Great work with insightful perspective on the effects of PARPi on BRCA1 resistant and deficient cells in co culture! The methodology is particularly well structured.”

The reviewers did however raise several useful points and questions, which we have thoroughly addressed in this improved revision. Reviewer 2 had several comments other aspects to consider, which differed from reviewers 1, 3 and 4, we have addressed these comments to the extent possible.

To address these main comments from all Reviewers, we have:

- Addressed all comments
- We have performed new experiments such as obtaining growth rates and performing a BRCA1 western.
- We have made substantial changes – based on the specific and constructive suggestions from Reviewers 1, 3, and 4.

We provide a full point-by-point response in the Response to Reviewers document. The updated results and revised text are marked in a tracked version of the manuscript. With these revisions, we believe that we have addressed the comments raised by the reviewers and have significantly improved the manuscript.

Kind regards,
Wayne Crismani on behalf of all authors

----

**Point by point responses.**

**Reviewer #1:**

This manuscript presents a competitive two-color co-culture assay designed to assess synthetic lethality and PARP inhibitor responses in BRCA1-deficient and resistant cell models. The system is well validated using both cancerous and non-cancerous isogenic cell lines, and the inclusion of resistance models (e.g., BRCA1 reversion, 53BP1-/-, SHLD1-/-) adds significant value to the findings.

The methods are clearly described and technically sound. The comparison to standard SRB assays provides useful context and highlights differences in IC50 values and dynamic responses between assay types. Flow cytometry-based quantification is appropriately used. The conclusions are well supported by the data.

We thank the reviewer for recognising the importance, utility, and quality of our method and manuscript.

A few minor revisions are recommended:
- Clarify the cause or implication of the discrepancy in IC50 values observed between the co-culture and SRB assays.

We discuss this further in the discussion on lines 348-357.

“Further, cell survival dynamics appear to be different when grown in co-culture demonstrated by the differing olaparib IC50’s between the competitive growth assay and the SRB assay. Additionally, IC50’s of the same cell line and drug can differ between competitive growth assays depending on the other cell line it is competing with (S2 table). This highlights how IC50’s calculated from any survival assay are context-dependent, including SRB and competitive growth assays, and caution must be taken not to generalise results from a single method..”

- Discuss the observed growth advantage of BRCA1-proficient cells at low PARPi doses, and whether this has biological or technical relevance.

We discuss this further on line 364-371.

“In the competitive growth assay, *BRCA1*-proficient cells sometimes appear to have an increase in survival at low concentrations of PARPi. The spike in survival seems to occur at the dose in which the *BRCA1*-deficient cells start to have a decrease in survival and where there is the greatest survival difference between the two cells lines. The proliferation in the *BRCA1*-proficient cells could be caused by reduced competition for nutrients due to the decrease in the *BRCA1*-deficient cells. Irrespective of the cause of the differential genotype-dependent response, it highlights the utility of this assay as a complement to survival assays that use a single genotype per well. .”

- Consider expanding briefly on the assay’s potential adaptability to additional tumor types or to 3D culture models, as this could broaden impact.

We have further discussed these points in the Future directions section of the Discussion (lines 438-444), which now reads:

“A competitive growth assay could also be applied in 3D cell cultures using fluorescent reporters to track the growth of different cell types (11). While 3D cultures are less high throughput than standard 2D culture, modelling competitive growth in 3D could provide a more accurate representation of in vivo cell survival and enhances in vitro pre-animal studies of new drug treatments. This assay is not limited to BRCA1 synthetic lethal relationships and could potentially be applied to any cell or tumour type that has a genetic vulnerability that leads to a selective pharmaceutical sensitivity in 2D or 3D.”

The manuscript is clearly written and meets standards for English usage. Data availability and ethical compliance are appropriately addressed. No concerns were identified regarding dual publication, ethics, or research integrity. The study does not involve dual use research of concern.

Recommendation: Accept with minor revisions.

**Reviewer #2:**

This manuscript lacks significant advancements in the described protocol and omits essential information regarding experimental sources.

While the authors have focused on the methodology, they have failed to provide sufficient details about the cell lines used. To complete this project, the authors must clarify the genetic and phenotypic characteristics of the cells or, at the very least, provide full references for both wild-type and mutant cell lines. Due to these omissions, many sections of the manuscript may lead to misleading conclusions.

We have added a new Supplementary table 1, which includes details of the cell line names, tissue, *BRCA1* status and variant information, relevant secondary mutations in PARPi resistance genes, and references.

Additionally, the manuscript's structure is confusing; the authors should separate the main text from the figures and figure legends. In conclusion, substantial revisions are needed—both in terms of additional data and structural organization—before this manuscript can be considered for publication.

We appreciate the comment regarding the structure of the manuscript. However, we have followed the PLOS ONE author guidelines for manuscript structure. Importantly, the manuscript’s structure and clarity were positively noted by all three other reviewers. For example:

Reviewer 1 stated that “The methods are clearly described and technically sound.” and “The conclusions are well supported by the data.”;

Reviewer 3 commented that “The study design is well executed with the data figure well assembled and easy to be understood. The statistical analysis is also clear”; and

Reviewer 4 described the methodology as “…particularly well structured” and praised the manuscript for offering a “Great work with insightful perspective...”

We are of course happy to further clarify any specific sections that may be unclear, but we believe the current structure is consistent with journal expectations and has been well received by the broader review panel.

**Reviewer #3:**

 The study design is well executed with the data figure well assembled and easy to be understood. The statistical analysis is also clear. The manuscript by virtue of its results is a solid candidate for publication if the authors can address my comments included in the attached review report.

Inhibition of PARP1/2 (PARPi) in homologous recombinant (HR) repair deficient cells is a classic example of synthetic lethality. Here, the authors focused on describing their competitive co-culture assay in the context of BRCA1-deficiency and PARPi. Specifically, they compared the fluorescent readouts in isogenic cell lines of BRCA1-deficient (mCherry) and BRCA1-proficient (eGFP) cells that are co-cultured in the presence of different concentrations of PARPi. Soetomo et al also applied their competitive assay in other previously reported PARPi resistant BRCA1 mutant/deficient clones (Azurite reporter). Indeed, BRCA1-deficient cells are sensitive to the PARPi tested (olaparib, niraparib, veliparib and talozoparib) while PARPi-resistant cell lines showed subtle differences compared to BRCA1-proficent cells. The study design is well executed with the data figure well assembled and easy to be understood. The statistical analysis is also clear.

**The manuscript by virtue of its results is a solid candidate for publication if the authors can address the following concerns:**

1. **Lack of western blot to confirm the presence or absence of mutant proteins.**

The authors generated SUM149.A22. that is a BRCA1+/- heterozygous cell line. Additionally, the study also utilized PARPi-resistant clones SUM149 B1.s* BRCA1^D80bp/-^ that restored the reading frame of the mutant allele in SUM149 cells. Both cell lines behave similarly in terms of PARPi resistance. Deletion of either 53BP1 or SHLD1 also restored PARPi resistance.

A western blot analysis for all the cell lines used (SUM149 and RPE) is helpful to determine the BRCA1 protein levels in the BRCA1-revertant and mutant cell lines used. After all, PARPi resistance is highly correlated with BRCA1 protein levels.

This is now included in Supplementary Figure 3. We have performed a western blot on cell lysates from the SUM149PT, SUM149 B1.s*, SUM149.A22 and both the RPE cell lines. The BRCA1 protein status is as expected where no BRCA1 is present in the RPE *BRCA1*^-/-^ and SUM149PT, but is expressed at comparable levels in in the RPE *BRCA1*^+/+^, SUM149 B1.s* and is restored in the newly developed SUM149.A22 cell line. We have updated the text with these important details on lines 234-237 and 296-297.

2. **How is the cell growth of the cells in the absence of PARPi considering that rapidly proliferating cells may be easier to be targeted with PARPi?**

I wonder if the revertant (SUM149.A22., SUM149 B1.s* )or PARPi-resistant (SUM149 BRCA1^-/-^ 53Bp1^-/-^ and SUM149 BRCA1^-/-^ SHLD1^-/-^ )cell lines have any significant growth differences with the parental cell that is BRCA1-deficient.

Since this is a competitive growth assay over the course of several days, it will be important to determine if the cell lines have similar proliferation rates or proliferation rate of the cells are not a determining factor in their assay. The authors also acknowledge how interpretation of competitive growth assay can be context specific, citing how cells with higher proliferative rates are more sensitive to cisplatin (Reference 23)

We have made a table with the SUM149 cell line doubling rates (S3 table). In this instance the doubling rates are all quite similar. We added this on lines 361-363.

“However, in this study, the SUM149 cell lines used had similar doubling rates, ranging from 26.5 h to 31.7 h (S3 table) demonstrating the competitive advantages are due to the genes mutated and not the rate of cell proliferation.”

3. **A table will be useful to summarize the IC50 values and AUCs with the appropriate statistical analysis of the different cell lines when treated with different PARPi.**

We have included this as Supplementary Table 2 and referred to the table on line 354.

We added to the text that:

“Further, IC50’s of the same cell line and drug can differ between competitive growth assays depending on the other cell line it is competing with (S2 table).”

In other words, IC50 values for a given cell line in a competitive growth assay depend on the cell line it is paired with and therefore should not be overinterpreted in isolation. We recognize this could be confusing to non-expert readers but agree that presenting the data in the supplementary material makes it available for advanced readers who wish to examine it in more detail.

4. **How would the sensitivity profile be if the 53BP1 KO cells are co-cultured with SHLD1 KO cells? Can this experiment be done?**

The 53BP1-RIF1-SHLD1 complex is important for restricting DSB resection and promoting NHEJ, especially in BRCA1-deficient cells.

Results from Figure 4D, 4F, S2B, S2C is quite interesting as marginal sensitivity was observed in SUM149 BRCA1-/- 53Bp1-/- and SUM149 BRCA1-/- SHLD1-/- compared to SUM149.A22. This piece of result was mentioned in line 289-296 but not well expanded. What could be the reason behind the disparity between 53BP1-KO and SHLD1-KO when treated with different PARPis.

This is an interesting point, and we have attempted to address this. We have attempted to generate these cell lines, as we did not have this colour combination available, but the results are not ready at the time of resubmission. As much as this is valid and interesting point, we do not think that these absence of these results detracts from the main aims of the manuscript.

5. Minor concerns:
a) Typo line 193-194 for figure 2 legend “ The IC50’s in the SRB assay were 0.11 μM and 9.01 μM for the BRCA1+/- and BRCA1+/- cells “ . It should be BRCA1-/- with 0.11 μM IC50?

This has been corrected to read “*BRCA1*^-/-^”

b) Word choice line 208-209 “ a phenomenon was observed” ; the word phenomenon is quite out of place in the long sentence.

This sentence has been revised to read “Consistent with observations in Fig 2A, BRCA1-proficient cells showed a growth advantage – evident as increased cell numbers – at low concentrations of olaparib and niraparib in the SUM149 background, and of olaparib, veliparib and niraparib in the RPE background.”

**Reviewer #4:**

2) For this comment, the n numbers is 8 in fig 2, 3 in fig 4 and have not been mentioned in fig 3. Perhaps the n numbers should have been consistent.
4)

We have added the ‘n’ numbers in each figure legend. Figure two has a greater number of technical replicates because while we were developing and validating the assay, we were gathering data to quantify variance. We established the technique and found that variance was low and therefore could proceed with n = 3 technical replicates as evidenced by the substantial difference in area under the curve for all PARPi in Figure 3D.

I have added in corrections for grammatical errors as well as structure of sentences used in the attached file. There are a few errors with the tenses as well which I have also pointed out.

Great work with insightful perspective on the effects of PARPi on BRCA1 resistant and deficient cells in co culture! The methodology is particularly well structured. Just a few minor suggestions from my end.

1) In the following section, I believe there should not be a comma if you are referring to a second complementary gene/pathway “By targeting a second, complementary gene or pathway that is synthetic lethal with the cancer-specific mutation, specific inhibitors can selectively kill cancer cells while sparing healthy cells lacking the mutation”

done

2) Line 39 – “PARPis” should be replaced with “PARPi” as the initially mentioned complete form of PARPi is “poly ADP-ribose polymerase inhibitors” (plural form)

Done

3) Line 51 – “complexity that can be more closely represent in vivo environment” – the highlighted word should be removed

Done

4) Line 61 – “In this present study,” replace highlighted word to “the” and change “competitive growth system that could be used” the highlighted word to “can” (if validated) or “may” (if requires further validation)

done

5) Line 62 – “PARPis” to “PARPi” (replace throughout the paper)

done

6) Line 100 – “After 48 h and 72 h after transfection,” change the highlighted word to “following”

Done

7) Line 116 – “72 h after the first dose of drug, cells were drugged again with the same concentrations” change to “Following a 72 h incubation period, cells were again exposed to the same concentration of the drug” (also add what condition the cells were incubated in – e.g room temp or incubator)

Done

8) Line 117 – “were fixed with with” remove duplicates

Done

9) Line 125 – “The data was normalized using by calculating - normalized percentage of survival” remove highlight word and add “the” to the highlighted dash inserted

Done

10) Line 128 – “A semilog curve was fitted to the data sets in which IC50’s were calculated from” replace with “A semi log curve was fitted to the data sets and IC50 values were calculated”

Done

11) Line 132 – “PARPis one day and 4 days” - keep format of days consistent – 1 day and 4 days/one day and four days

Done

12) Line 136 “The data was normalised using by calculating - normalized percentage of survival” remove highlighted word, add “the” to the highlighted dash inserted

Done

13) Line 142-144 – “The SUM149PT cell line was originally derived from a breast adenocarcinoma and 143 contains a homozygous BRCA1 pathogenic variant, c.2169delT, which results in a frameshift 144 that leads to a premature stop codon (p.P724Lfs*12).” – part of the section uses past tense while part of it uses present tense. I would suggest using past tense as the rest of the paragraph is also in past tense and change “contains” to “contained”, “results” to “resulted” and “leads” to “led”

Done

14) Line 156 – “The schematics of each assay is” change to “are”

Done

15) Line 163 – “Fig 1. Schematic of the competitive growth assay vs SRB assay” and a full stop at the end

Done

16) Line 164 in Fig 1 legend – “in the small wells of a microplate” change this to the actual 96-well plate, this sound informal

Done

17) Fig 1 legend – present tense used again while the rest of paper is in past tense.

Done, changed to past tense.

18) Fig 2 legend – “8 technical replicates were performed, and 192 error bars represent standard error of the mean” – I would recommend adding this section as the last sentences of the figure legend where you could write “Data is plotted as mean ± SEM where n = 8 for each treatment group”

Note: I am wondering why some of the data in this figure for both strains do not have error bars?

Done.

Some of the data points have such low SEM that the symbol is larger than the error bars, this is why they are not visible on the graph.

19) Fig 3 – “. A) SUM149PT BRCA1-/- vs SUM149.A22 BRCA1+/- 216 cells” could mention that is shows cell survivial. Same comment for “C) RPE BRCA1-/- vs RPE BRCA1+/+ 218 .”

Note: figures in (A) are showing error bars while figures in (C) are not. Could also mention the n number and whether the data represents mean +- SEM.

Done.

20) Figure 4 – same comment about the error bars – would recommend standardizing the error bars across figures.

Similar to comment 18, the error bars are plotted but they are too small to see on some data points.

21) Line 282 – “50% survival observed - BRCA1 negative cells” - add “for” in the highlighted dash position

Done

22) Line 283-284 – “near-complete cells death” - make it singular

Done
